# The Impact of Bisphenol A on Thyroid Function in Neonates and Children: A Systematic Review of the Literature

**DOI:** 10.3390/nu14010168

**Published:** 2021-12-30

**Authors:** Diamanto Koutaki, George Paltoglou, Aikaterini Vourdoumpa, Evangelia Charmandari

**Affiliations:** 1 Division of Endocrinology, Metabolism and Diabetes, First Department of Pediatrics, National and Kapodistrian University of Athens Medical School, ‘Aghia Sophia’ Children’s Hospital, 11527 Athens, Greece; dkoutaki@med.uoa.gr (D.K.); gpaltoglou@gmail.com (G.P.); katvourdouba@gmail.com (A.V.); 2 Division of Endocrinology and Metabolism, Center of Clinical, Experimental Surgery and Translational Research, Biomedical Research Foundation of the Academy of Athens, 11527 Athens, Greece

**Keywords:** bisphenol, BPA, thyroid, children, neonates, TSH, T4, neurodevelopment

## Abstract

Background: Bisphenol A (BPA) is an endocrine-disrupting chemical widely used in plastic products that may have an adverse effect on several physiologic functions in children. The aim of this systematic review is to summarize the current knowledge of the impact of BPA concentrations on thyroid function in neonates, children, and adolescents. Methods: A systematic search of Medline, Scopus, Clinical Trials.gov, Cochrane Central Register of Controlled Trials CENTRAL, and Google Scholar databases according to PRISMA guidelines was performed. Only case–control, cross-sectional, and cohort studies that assessed the relationship between Bisphenol A and thyroid function in neonates and children aged <18 years were included. Initially, 102 articles were assessed, which were restricted to 73 articles after exclusion of duplicates. A total of 73 articles were assessed by two independent researchers based on the title/abstract and the predetermined inclusion and exclusion criteria. According to the eligibility criteria, 18 full-text articles were selected for further assessment. Finally, 12 full-text articles were included in the present systematic review. Results: The presented studies offer data that suggest a negative correlation of BPA concentrations with TSH in children, a gender-specific manner of action, and a potential effect on proper neurodevelopment. However, the results are inconclusive with respect to specific thyroid hormone concentrations and the effect on thyroid autoimmunity. Conclusion: The potential negative effect of BPA in the developing thyroid gland of children that may affect proper neurodevelopment, suggesting the need to focus future research on designing studies that elucidate the underlying mechanisms and the effects of BPA in thyroid function in early life.

## 1. Introduction

In recent decades, accumulating evidence has demonstrated the impact of environmental endocrine-disrupting chemicals (EDCs) on human health [1]. These substances are defined by the U.S. Environmental Protection Agency (EPA) as “an exogenous agent that interferes with synthesis, secretion, transport, metabolism, binding action, or elimination of natural blood-borne hormones that are present in the body and are responsible for homeostasis, reproduction, and developmental process” [1]. This definition is further detailed by the Word Health Organization as “an exogenous substance or mixture that alters the function(s) of the endocrine system and consequently causes adverse effects in an intact organism, or its progeny, or (sub) populations” [2]. Endocrine-disrupting chemicals are a heterogeneous group and can be detected in a wide spectrum of daily used products. Bisphenol A (BPA, 4,4′-isopropylidenediphenol) is a monomer EDC used in the manufacture of poly-carbonate plastics and epoxy resins [3]. It is also highly used in consumer products, such as baby feeding bottles, protective coatings inside food containers, and water supply pipes, as an additive that can improve the properties of plastics [4,5]. The exposure is ubiquitous given that BPA is one of the chemical compounds used in massive amounts in the production of synthetic polymers and thermal paper, with an annual production of at least 8 million tons throughout the world, while the European Union has legally restricted the use of BPA in thermal paper in concentration equal to or greater than 0.02% by weight [5,6]. Oral use is considered to be the primary route of contamination, followed by the dermal route; however, there are also reports suggesting the possibility of indoor air contamination [7]. In addition to adults, children are also highly exposed to it as well [8]. In a large study of 2517 participants conducted by the Centers for Disease Control, children had significantly higher concentrations of BPA compared with adults, which, along with the reported presence of BPA in amniotic fluid, suggest an early exposure to BPA [9,10]. 

As an EDC, BPA has been associated with numerous health consequences affecting almost every organ, including the function of the reproductive and the cardiovascular system, the cognitive and behavioral development, and the pathogenesis of obesity [11,12,13]. Moreover, animal and human studies provide evidence of this particular phenol interfering with the pituitary–thyroid axis [14]. Mechanistically, BPA acts on various molecular pathways in many tissues. It can interact with numerous receptors, including nuclear and membrane-bound receptors, as well as transcription factors, and it may also induce epigenetic changes [15,16,17]. The more thoroughly described interaction of BPA is that with nuclear steroid estrogen receptors alpha (ERα) and beta (ERβ) [18]. Bisphenol A also binds with a strong affinity to estrogen-related receptors gamma (ERRγ) [19]. After binding with the receptor, BPA can act like an estrogen, albeit with a weaker activity than 17-beta estradiol (E2) [20]. Moreover, its estrogenic properties are influenced by the ratio of estrogen receptor (ER) subtypes in a particular cell/tissue, suggesting differential responsiveness [18]. BPA also displays anti-androgenic properties, being a competitive antagonist to 5α-dihydrotestosterone (DHT) for binding to the androgen receptor (AR) [21,22]. Of note, some studies have indicated that BPA can act as an agonist of the glucocorticoid receptor (GR) [23,24]. 

Accumulating evidence suggests a role of BPA in transcription factor physiology. Firstly, BPA can induce peroxisome proliferator-activated receptor γ (PPARγ) and thus adipogenesis in several (animal) models [25]. The expression of genes essential for proper growth and development, such as *HOXC1* and *HOXC6*, *WNT5A*, *FZD5*, *TGFβ2*, and *SOCS2*, is also affected by this BPA [26]. Furthermore, it can cause epigenetic changes, such as altered methylation and interpretation with the expression of multiple microRNAs (miRNAs) [27,28,29].

Bisphenol A can perturb normal thyroid function in many ways, including interaction of cellular signaling and gene expression (transcription) [30]. The majority of in vitro studies suggest that BPA binds to thyroid hormone receptors (TR)-alpha and TR-beta, thereby antagonizing the action of triiodothyronine (T3) and suppressing its transcriptional activity [30,31,32,33]. Some studies indicated that BPA can act as an agonist to the thyroid receptor, especially in very low doses, that could potentially suggest a non-monotonic dose response (NMDR) that could go against predictable or typical dose–response patterns [34,35]. Furthermore, a low dose of BPA suppresses T3-induced transcription by recruiting nuclear receptor co-repressors (N-CoRs) to the thyroid receptor [30]. The recruitment of N-CoR to TR-beta1 by BPA in a low-dose exposure is mediated through a non-genomic mechanism by β3 integrin/c-Src/MAPK/TR-beta1 pathways [36]. When BPA is detected in usual concentrations in human serum, there is no significant competition for binding with thyroid hormone transport proteins (human serum albumin (HSA), transthyretin (TTR), and thyroxine-binding globulin (TBG)) despite its structural similarity to thyroxine (T4) and T3 [37]. At the gene level, BPA can interact with the expression of numerous genes affecting thyroid homeostasis, including genes associated with inhibition of different types of deiodinases [38,39]. Moreover, it disrupts the expression of genes involved in thyroid hormones synthesis (tshβ, Slc5a5, Tpo, and Tgo, Pax8, Foxe1, and Nkx2-1) through a direct effect on thyroid follicular cells but not on the activity of thyroid peroxidase [39,40,41]. Finally, it inhibits sodium/iodide symporter (NIS)-mediated iodide uptake at the post-translational level [39].

Results from a cross-sectional study on a human cohort from the National Health and Nutrition Examination Survey (NHANES) study in 1346 adults indicated an inverse relationship between urinary BPA and total thyroxine and thyroid-stimulating hormone (TSH) [42]. This is particularly worrying given the fact that normal thyroid function is important for the developing brain of the fetus [43]. It should be noted that these substances can cross the placenta and affect the developing fetus [44]. In addition, there is evidence that even mild maternal hypothyroxinemia during gestation can alter fetal neurodevelopment, behavior, and cognition in the offspring [43,45,46]. Apart from short-term health risks, it still remains unclear whether this substance can affect the thyroid function later in life, given the hypothesis of the developmental origins of health and disease (DOHaD) [47]. Although BPA may have long-term adverse effects on several functions and may lead to trans-generational changes in children’s behavior, it is not clear whether it affects thyroid function [48,49].

Epidemiological studies reveal a possible effect of BPA on thyroid function in adult populations; however, few data exist regarding its effect in children and adolescents. The aim of this systematic review is to summarize the current knowledge on adverse effects of BPA on the pituitary–thyroid axis of neonates, children, and adolescents.

## 2. Materials and Methods

### 2.1. Study Design

The present study was conducted according to the Preferred Reporting Items for Systematic Reviews and Meta-Analyses (PRISMA) protocol (supplementary material) [50]. The study protocol included the following consecutive stages: (i) primary research of the systematic literature using search engines; (ii) selection of studies to be included according to our inclusion and exclusion criteria; (iii) data extraction; and (iv) data synthesis and analysis. 

### 2.2. Eligibility Criteria

The eligibility criteria were predetermined by the authors. In order to prevent language bias, our search was not limited by language. Additionally, there was no date or country restriction. We included case–control, cross-sectional, and cohort studies that assessed the relationship between BPA and thyroid function in neonates and children younger than 18 years. Reviews, letters, abstracts, case reports, expert opinions, and in vitro or animal experiments were excluded from our data selection. All inclusion and exclusion criteria in terms of study type, participants, language, geographic location, and outcomes are described in Table 1.

### 2.3. Literature Search

Two independent reviewers (DK) and (GP) used the Medline (2005–2021), Scopus (2005–2020), Clinical Trials.gov (2008–2021), Cochrane Central Register of Controlled Trials CENTRAL (2011–2021), and Google Scholar (2005–2021) search engines in primary search prior to June 2021. 

Our search strategy included the terms: “thyroid*” [All Fields] AND (“bisphenol*” [All Fields] OR “bisphenol a” [All Fields] OR “BPA” [All Fields]) AND (“child*” [All Fields] OR “adolescen*” [All Fields] OR “neonat*” [All Fields]). Articles together with reference lists from included studies were retrieved. 

### 2.4. Study Selection

After the initial search, all the obtained documents were screened independently by the two reviewers (DK and GP). The two reviewers used Mendeley software to exclude all duplicates and examined eligibility for the studies based on the relevance of their title and abstract to the subject (first eligibility check). For the relevant articles, full text was assessed based on the inclusion and exclusion criteria (second eligibility check). During the whole procedure, each reviewer remained blinded to the other investigator’s selection. When conflict between the two authors occurred, it was resolved through a third author (AV).

**Figure 1:**
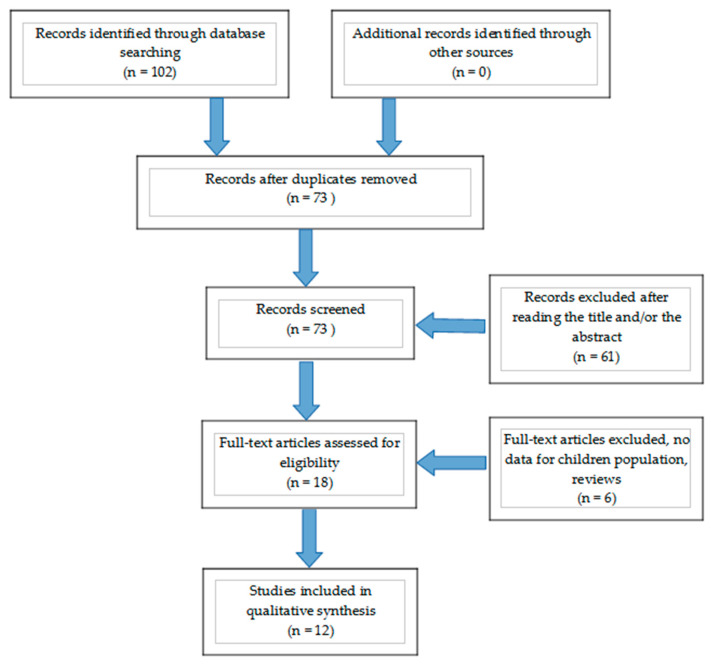
Flow chart describing the step-wise selection (identification, screening, eligibility, inclusion) of the studies included in the qualitative synthesis.

Following the terms listed above, the electronic database search resulted in 102 articles, which were then checked for duplicates. After exclusion of duplicates, our list was restricted to 73 articles. No additional records were from other sources (manual research, reference lists of other papers). A total of 73 articles were thoroughly assessed by two independent researchers based on title/abstract and the inclusion and exclusion criteria. According to the eligibility criteria, 18 full-text articles remained for further assessment. Among these full-text articles, 6 were excluded for the following reasons: wrong study type, namely reviews/systematic reviews, or information that did not meet the eligibility criteria and the purpose of this systematic review (third step). Finally, 12 full-text articles were considered eligible and were included in our qualitative synthesis. The study selection procedure is detailed in the respective flow chart (Figure 1). 

### 2.5. Data Extraction

Data extraction was conducted using a standardized excel data extraction form, and the process was regulated by the same two reviewers (DK and GP), who worked blinded and separately. The following data items were extracted: reference of the article (Author, Year), study type (case–controls, case series, cohort studies, randomized controlled trials), sample characteristics and concentrations of BPA and thyroid hormones, and time and method of BPA measurement.

## 3. Results

In 2013, the Center for the Health Assessment of Mothers and Children of Salinas study (CHAMACOS) investigated in a Mexican-American population the impact of maternal BPA exposure during the first and second half of pregnancy on offspring’s TSH [51]. The researchers revealed a statistically significant (*p* = 0.01) inverse relationship in male neonates (−9.9% per log2 unit; 95% CI: −15.9%, −3.5%) but not in females (4.4% for every doubling in average BPA; 95% CI: −2.4%, 11.7). Furthermore, among boys, the association was stronger when maternal BPA urinary concentrations were measured in the third trimester of gestation, suggesting a developmental window of susceptibility. Moreover, in the Health Outcomes and Measures of the Environment (HOME) Study, a 10-fold increase in mean BPA was associated with lower cord TSH concentrations in girls (percent change = −36.0%; 95%(CI): −58.4, −1.7%) [52]. Similar to the CHAMACOS study, a more evident effect of BPA was observed during late gestation. A suggestive positive association between maternal urinary BPA and TT3 concentrations among newborn females was also observed (*p* = 0.11). The inverse BPA–TSH relation among girls was stronger but less precise among iodine deficient mothers. A negative correlation between BPA concentrations and TSH (r = −0.25, *p* = 0.077) was observed in another small cohort of 53 newborn males similar to previous studies [53].

Fen Li et al. evaluated the effect of the exposure to BPA during the first trimester of gestation on children’s neurobehavioral development at 2 years and 4 years. A significant inverse relationship between BPA and TSH concentrations in both boys and girls was noted (βhighest = −1.91, 95%CI: −3.32, −0.50; for boys, βhighest = −1.89, 95%CI: −3.70, −0.50; for girls, βmiddle = −2.34, 95%CI: −4.61, −0.07). Children with the middle or highest tertile of BPA concentration also had lower TT3 (βhighest = −0.05, 95%CI: −0.10, −0.01), FT3 (βhighest = −0.12, 95%CI: −0.22, −0.02; βmiddle = −0.11, 95%CI: −0.21, −0.01). Therefore, it was suggested that prenatal BPA exposure may be associated with increased risk of neurobehavioral problems, especially in boys [54].

The same strategy was followed in a longitudinal cohort of Asian population, where maternal urinary BPA concentrations were associated with increased risk of total difficulties in children aged 10 years in a gender-specific manner, with boys being more vulnerable. Concerning the thyroid hormones, BPA levels were significantly correlated with 1.00% (95%CI: 0.20%, 1.92%) increases in cord serum FT4 concentrations [55].

However, some studies reported no association between BPA and thyroid hormone levels, such as the Hokaido study (Pinteraction = 0.819 for TSH and P interaction = 0.969 for FT4) [56], and a cross-sectional study in which pregnant women were stratified according to BMI (BMI < 18.5, 18.5–22.9 or >23 kg/m^2^) [57]. No association was observed between prenatal urinary BPA concentration and cord serum FT4, FT3, TSH concentrations of neonates, as well as the odds of TPO-Ab positivity (>5.61 IU/mL). However, in mothers with higher BMI and higher BPA exposure, cord serum FT4 was 2.96 (95% CI 0.12–5.80) pmol/L higher in male newborns and 2.22 (95% CI 0.67–3.78) pmol/L higher in female newborns compared to those in the low tertile of BPA levels. No significant association was detected between cord blood concentrations of T4, TSH, SPINA-GT (thyroid incretory capacity), TSH Index (TSHI), standardized TSHI (sTSHI) concentrations of infants, and cord blood BPA in both genders included in another report [58]. Finally, in a recent publication, BPA was measured twice (during gestation and at 6 years); however, there was not any significant association except a relationship between prenatal BPA and T3 only when stratified by sex [59].

Only one study reports a positive association between BPA and TSH [60]. In this cohort, urine samples for detection of the xenobiotic were acquired during early, middle, and late pregnancy (<18, 18–25, >25 weeks gestational age) and thyroid function was evaluated both short-term after birth and long-term in childhood (5.9 years). A higher BPA level during late pregnancy was associated with a higher cord blood TSH in newborns, especially in females, but not in early or mid-pregnancy (β (95% CI): 0.04 (0.007 to 0.07)) and a lower FT4 concentration in childhood (β (95% CI): −0.11 (−0.21 to −0.01).

Limited data exist concerning the effects of BPA on thyroid volume, the presence of thyroid nodules and Hashimoto thyroiditis. Urinary BPA concentration was negatively associated with thyroid volume (assessed by thyroid ultrasonography) in 718 children aged 9–11 years (β = −0.033, 95% CI: −0.053, −0.013) and the risk for multiple nodules but not with the risk of solitary nodules (n = 100, OR = 0.78; 95% CI: 0.63, 0.97) [61]. Specifically, each log unit increase in BPA level was associated with a 0.088 SD unit decrease in thyroid volume. The median thyroid volume of participants was 3.14 mL and was similar for boys (3.05 mL) and girls (3.21 mL). The authors attributed this decrease in thyroid volume to a possible negative correlation between BPA and TSH.

Sur et al. examined the relationship between BPA and Hashimoto thyroiditis (HT) (diagnosis was made by i. increased plasma concentrations of thyroid peroxidase auto-antibodies (anti-TPO); ii. heterogeneous echo-texture in thyroidal ultrasound) [62]. Urinary BPA levels were not significantly different in the HT group and the control group. There was a negative correlation between BPA level and FT4 concentrations (r = −0.483, *p* < 0.02), and no correlation was observed between urinary BPA concentrations and anti-TPO levels (*p* = 0.063).

The details of the included studies are presented in Table 2, Table 3 and Table 4. More specifically, the characteristics of each study concerning the design, number of participants, sample of thyroid hormones, BPA sample, and time of BPA measurement are presented in Table 2. Mean/median values for each study of BPA and thyroid hormones (TSH, FT4, TT4, Ft3, TT3) are presented in Table 3. The statistically significant odds ratios (OR), correlations (r), or logistic regression models (betas) of the association of BPA with TSH, T4 and T3 where applicable are presented in Table 4.

## 4. Discussion

Bisphenol A (BPA) preserves endocrine disruptive properties and can interact with almost every human system. Humans are ubiquitously exposed daily to plastic products in the modern world [63]. An expanding number of studies address the impact of BPA in thyroid function in recent years [14,64]. Many researchers have tried to evaluate the trace of xenobiotic exposure, such as BPA, on thyroid function and specifically in the pediatric population.

We found that many of the collected data in children suggest a negative correlation of BPA levels with TSH; however, the results are inconclusive with respect to the thyroid hormone concentrations. Similarly, in large adult studies, BPA was associated inversely with TSH concentrations [42,65,66]. This observation can be attributed to the structural similarity of BPA to the thyroid hormones, thyroxine (T4), and triiodothyronine (T3), and its action as an antagonist or agonist of the thyroid receptor [34,35]. Experimental data indicated that BPA could suppress the TRH-induced release of both TSH and prolactin [67]. The suppression of TSH secretion from the pituitary was independent of the thyroid hormone negative feedback and the estrogenic activity of BPA [67]. Moreover, this association is in accordance with the reported decrease in thyroid volume [61]. An exposure to high concentrations of BPA may result in lower concentrations of TSH and consequently to a decreased volume of the thyroid gland [61]. It is well described that TSH stimulates hypertrophy and hyperplasia of the thyroid follicular cells, leading to the enlargement of the thyroid, while TSH also increases blood flow to the thyroid gland [68]. Although embryogenesis of the thyroid is reported to be independent of TSH until the 14th week of gestation [69], it can be hypothesized that lower concentrations of fetal TSH during late gestation would lead to decreased volume of thyroid gland given the loss of TSH trophic effect, it has been reported in autopsy findings in patients with congenital hypopituitarism [70].

Several studies support the concept that BPA can act in a gender-specific manner [51,52] because of its ability to interact with estrogen and androgen receptors [16]. This differential responsiveness of BPA can be shown when assessing its estrogenic properties and how they are influenced by the ratio of ER subtypes in a particular cell/tissue [18]. Rodent studies have also assessed a gender-specific effect of BPA with contradicting results [71,72]. While one experimental study found that exposure to BPA during pregnancy and postpartum was related to alterations in FT4 among male rodents in a dose-dependent manner [72], another showed similar thyroid hormone responses to BPA in both male and female rodents [71]. A gender-specific impact of BPA apart was also observed on fetal organ weights, steroid profiles, and growth trajectories [73]. Furthermore, several studies evaluated the effect of BPA on the thyroid function of neonates, given that thyroid hormones are essential for proper brain development during early life stages and that BPA can cross the placenta [74,75]. In addition, in utero exposure can result in toxicity as fetuses lack the enzyme UDP-glucuronosyltransferase (UDPGT) required to metabolize BPA and thus are more sensitive [76]. The possibility of a vulnerable time window during perinatal exposure and offspring’s thyroid function has also been assessed as it has been shown that exposure at late gestation prior to birth can have more evident effects on thyroid function [51,52]. This result is in line with thyroid gland organogenesis, as fetal thyroid gland becomes mature and begins to produce thyroid hormones after week 20 of gestation; therefore, BPA exposure after the second trimester of pregnancy could have a greater effect on fetal thyroid gland function [77]. However, this differential effect of BPA on thyroid function after the second trimester can be confounded by the potentially shorter period between the measurement of BPA and thyroid hormones at birth.

Accumulating evidence suggests that the increased prevalence of autoimmunity in industrial areas may be attributed only to the exposure to “endocrine disrupting chemicals” [78]. BPA has been linked to the pathophysiology of autoimmune diseases [79]. Perinatal exposure to BPA predisposes mouse pups to the development of asthma [80]. Autoimmune hypothyroidism in childhood has an estimated prevalence of 1% to 2% and is characterized by gender dimorphism with a 4:1 female predominance [81]. When thyroid autoimmunity was assessed in children and adolescents, no significant correlation of anti-TPO antibodies and heterogeneous echo-texture in thyroidal ultrasound with BPA was found [57,62]. However, another study conducted in 2361 adults, aged >15 years, found that BPA was independently associated with positive anti-TPO antibodies [82]. A variety of mechanisms by which BPA may be a triggering compound to autoimmunity have been described, including its impact on hyperprolactinemia, estrogenic immune signaling, cytokine expression, and activation of macrophages, cytochrome P450 enzyme disruption, molecular mimicry to triiodothyronine, and immunoglobulin pathophysiology with increased production of IgA and IgG2a [79].

This disruption of the thyroid hormones in early life may be associated with neurobehavioral pathology later in life, especially in boys [54,55]. Data from observational studies indicate an association between both prenatal and childhood exposure to BPA and adverse behavioral outcomes in children with higher levels of anxiety, depression, and hyperactivity [83]. In vitro data suggest that BPA inhibits the TR-mediated differentiation of oligodendrocyte precursor cells [84] and perinatal exposure to low concentration of BPA inhibits synaptogenesis and affects synaptic structural modification after birth [85]. Furthermore, in Xenopus laevis brain, BPA interfered with thyroid hormone signaling and affected TH-dependent brain development [86]. Finally, the gender-specific associations between phenol exposures and children’s behaviors may be partly attributed to the fact that maternal phenols exposure may disrupt the development of sexually dimorphic brain structures of the fetus [87].

In summary, the presented studies show that early-life exposure to BPA during the fetal and perinatal period and in childhood can program molecular changes that can manifest in adulthood and pass on to subsequent generations [88]. Fetuses are extremely susceptible to environmental stress and disruptors due to their innate immaturity, while the reported effects of BPA on neurobehavioral development may be partly due to disruption of thyroid function. Maintaining normal thyroid function during the perinatal period, infancy, and childhood is extremely important for ensuring normal mental development and neurobehavior in life [89]. The present study has a number of limitations. First, due to its design, we are unable to make any conclusions regarding causation in the associations, particularly given methodological variations in the presented studies, despite the effort for this variation to be minimized. This is further hampered by the fact that BPA affects thyroid function via various physiological mechanisms, interacting in a complex and intricate manner with the molecular pathways involved. Finally, it should be noted that BPA has a relatively short half-life of 6 h in humans, as it is glucuronidated in the liver and excreted in urine, so a single spot urine sample may not be a reliable measurement [14,90]. The main strengths of our study rely in its methodology, which strictly follows PRISMA guidelines, and its novelty, as to our knowledge, this is the first systematic review summarizing the possible effects of BPA on thyroid function of children.

In conclusion, the above data suggest a negative association of BPA with TSH concentrations in children along with a gender-specific manner of action that may affect proper neurodevelopment. The presented potential impact of BPA in the developing thyroid gland of children, particularly in late gestation, reinforces the advice to limit use of BPA-contaminated products such as plastic baby bottles. Further prospectively designed studies are needed to better elucidate the association between endocrine disruptors such as BPA and the mechanisms that perturb thyroid function and organogenesis.

## Figures and Tables

**Table 1:** Inclusion and exclusion criteria.

Inclusion Criteria	Exclusion Criteria
Age < 18 years Studies from any geographic location Any publication date Observational and interventional studies Any language Assessing the relationship between BPA (maternal or neonatal) and thyroid function in neonates, children, and adolescents	Age ≥ 18 years Reviews, editorials, abstracts, case–controls, expert opinions In vitro or animal experiments

**Table 2:** General characteristics of included studies.

Authors	Number of Children	Method of BPA Measurement	BPA Sample	TSH Sample	Study Characteristics	Time of BPA Measurement
Brucker-Davis et al. (2011) [53]	53 (only boys)	Chromatography coupled with mass spectrometry	Cord blood	Cord blood	Prospective–Cohort	At birth
Chevrier et al. (2013) [51]	364	Online solid-phase extraction coupled with isotope dilution–high-performance liquid chromatography–negative ion–atmospheric pressure chemical ionization tandem mass spectrometry	Urine	Blood spots	Cohort	12.4 ± 3.8 and 26.2 ± 2.2 weeks of gestation
Romano et al. (2015) [52]	249	Online solid-phase extraction coupled to high-performance liquid chromatography-isotope dilution tandem mass spectrometry	Urine	Cord blood	Prospective	16 (13.0–20.9) and 26 (23.1–34.6) weeks of gestation
Wang et al. (2015) [61]	718	Ultra-performance liquid chromatography coupled with tandem mass spectrometry	Urine		Cross sectional	9–11 years old
Minatoya et al. (2017) [56]	283	Isotope dilution liquid chromatography-tandem mass spectrometry (ID-LC/MS/MS)	Cord blood	Heel-prick blood sample	Prospective–Cohort	At birth
Sanlidag et al. (2018) [58]	88	Sandwich enzyme—linked Immunosorbent assays (ELISAs)	Cord blood	Cord blood	Cross-sectional	At birth
Sur et al. (2019) [62]	29 and 29 control group	High-pressure liquid chromatography (HPLC)	Urine		Case-control	8–16 years old
Wang et al. (2020) [57]	398	Liquid chromatography tandem mass spectrometry (HPLC-MS/MS)	Urine	Cord blood	Cohort	Late pregnancy (38.8 ± 1.1 weeks of gestation)
Fen Li et al. (2020) [54]	348	High-performance liquid chromatography (HPLC)	Urine	Cord blood	Prospective	12–16 weeks of gestation
Guo et al. (2020) [55]	386	Gas chromatography tandem mass spectrometry	Urine	Cord blood	Prospective-Cohort	At birth
Derakhshan et al. (2020) [60]	853 neonates	Liquid–liquid extraction (LLE) followed by enzymatic deconjugation of the glucuronidated bisphenols accompanied by high performance liquid chromatography electrospray ionization-tandem mass spectrometry (HPLC-ESI-MS/MS)	Urine	Cord blood	Prospective	<18, 18–25, >25 weeks of gestation
	882 children		Urine	Serum		
Jang et al., 2021 [59]	574	High-performance liquid chromatography-tandem mass spectrometry	Urine	Blood sample	Prospective–Cohort	20 weeks of gestation and 6 years old children

Abbreviations: TSH: thyroid-stimulating hormone; BPA: bisphenol A.

**Table 3:** Mean concentrations of BPA and thyroid hormones.

Authors	TSH	T3	T4	BPA	Other
Brucker-Davis et al. (2011) [53]	Mean: 7.67 mIU/L (SD: 5.04) R = −0.25, *p* = 0.077 ↓	Mean: FT3 = 2.04 pmol/L (SD: 0.44) (-)	Mean: FT4 = 13.04 (pmol/L) (SD: 1.54) (-)	Median: 0.9 ng/mL (Range: 0.2–3.3)	
Chevrier et al. (2013) [51]	GM: 5.6 mIU/L (GSD:1.8) ↓ (boys)			Median: 1.2 mg/g creatinine (IQR: 0.8–1.9)	
Romano et al. (2015) [52]	Mean: 7.2 mIU/L (95%CI: 6.7, 7.8) ↓ (girls)	Mean ± SD FT3 = 1.7 ± 0.3 pg/mL TT3 = 52 ± 19 ng/dL ↑	Mean ± SD FT4 = 1.0 ± 0.2 ng/dL TT4 = 9.6 ± 1.8 mg/dL (-)	Median BPA (mg/g Cr) = 2.2 (IQR:1.5–3.4)	
Wang et al. (2015) [61]				Median: 2.45 (IQR: 1.09–5.97) μg/g creatinine	Median thyroid volume: 3.14 mL (IQR: 2.44–4.11)
Minatoya et al. (2017) [56]	Median Boys (127): 2.2 (IQR: 1.2–4.0) Median Girls (156): 2.2 (IQR1.4–3.9) (μU/mL) (-)		Median Boys (127): FT4 = 2.0 (IQR: 1.9–2.3) (ng/dl) Median Girls (156): FT4 = 2.0 (IQR: 1.8–2.3) (ng/dl) (-)	Mean ± SD: 0.057 ± 0.036 ng/ml	
Sanlidag et al. (2018) [58]	Mean ± SD: 4.85 ± 1.73 uIu/mL (-)		Mean ± SD: FT4 = 0.95 ± 0.2 ng/dL (-)	Mean ± SD: 4.934 ± 2.33 ng/mL	SPINA-GT sTSHI
Sur et al. (2019) [62]				Mean ± SEM: 7.72 ± 1.74 in control group and Mean ± SEM:7.31 ± 1.46 in HT group (μg/g creatinine) (-)	Hashimoto
Wang et al. (2020) [57]	GM: 5.48 (95%CI: 5.22, 5.77) mIU/L (-)	FT3 (pmol/L) Mean ± SD: Low tertile: 1.87 ± 0.34 Medium tertile: 1.81 ± 0.32 High tertile: 1.85 ± 0.38 (-)	FT4 (pmol/L) Mean ± SD: Low tertile: 13.75 ± 1.45 Medium tertile: 13.84 ± 1.35 High tertile: 13.82 ± 1.38 (-)	GM: 1.32 ng/mL (95%CI: 1.17–1.49)	Positivity of TPO-AbMean ± SD: Low tertile: 11 (8.3) Medium tertile: 15 (11.4) High tertile: 8 (5.9) (-)
Fen Li et al. (2020) [54]	Median: 6.43 μIU/L ↓	Median: TT3 = 0.85 nmol/L Median: FT3 = 1.78 pmol/L ↓	Median: FT4 = 14.18 pmol/L Median: TT4 = 92.90 nmol/L	Maternal median: 1.30 μg/g Cr Children’s median: 0.51 μg/g Cr	Neuro-development
Guo et al. (2020) [55]	Median: 6.88 pmol/L (IQR: 4.93 −9.49) (-)	Median: FT3 = 2.5 (IQR:2.3–2.7) pmol/L Median: TT3 = 0.89 (IQR:0.78–1.00) nmol/L (-)	Median: FT4 = 16.0 (IQR: 14.8–17.2) pmol/L Median: TT4 = 126 (IQR: 110–140) nmol/L ↑	Maternal Median: 1.75 μg/L (IQR: 0.60–16.1) Children Median: 1.29 μg/L (IQR:0.56–2.40)	Median: TPOAb = 14.7 (IQR: 12.5–17.4) IU/mL
Derakhshan et al. (2020) [60]	Median (95% range) Newborn: 9.57 mU/L (3.13–34.7) ↑ Child: 2.33 mU/L (0.92–4.87)		Median (95% range) Newborn FT4 = 20.6 pmol/L (14.8–31.0) Child FT4 = 16.8 pmol/L(13.7–20.9) ↓	Median (95% range) Early pregnancy: 1.61(<LOD-21.0) ng/mL Middle pregnancy: 1.47(<LOD-21.2) ng/mL Late pregnancy: 1.65(<LOD-20.5) ng/ml	
Jang et al. 2021 [59]	Mean ± SD: 2.55 ± 1.36 μIU/mL (-)	Mean ± SD: TT3 = 148.00 ± 18.47 ng/dL	Mean ± SD: FT4 = 1.15 ± 0.11 ng/dL (-)	Mean ± SD: Children 2.73 ± 7.15 μg/L Mean ± SD: Prenatal 2.15 ± 2.85 μg/g Cr	

Abbreviations: TSH: thyroid-stimulating hormone; BPA: bisphenol A; T4: thyroxine (T4); T3: Triiodothyronine, SD: Standard deviation, IQR: Interquartile Range, GM: Geometric Mean, GSD: Geometric Standard Deviation, SEM: Standard Error of the mean, CI: Confidence Interval, LOD: Limit of detection. ↓: negative association, ↑: positive association, (-) no significant association.

**Table 4:** Statistically significant correlations (r), odds ratios (OR), or logistic regression models (betas) of the association of BPA with TSH, T4, and T3.

TSH	
Brucker-Davis et al. (2011) [53]	Negative correlation between BPA concentrations and TSH (r = −0.25, *p* = 0.077)
Chevrier et al. (2013) [51]	Inverse relationship between maternal BPA concentrations and TSH in boys (−9.9% per log2 unit; 95% CI: −15.9%, −3.5%)
Romano et al. (2015) [52]	Inverse relationship between maternal BPA concentrations and TSH in girls (percent change = −36.0%; 95%(CI): −58.4, −1.7%)
Fen Li et al. (2020) [54]	Significant inverse relationship between BPA and TSH concentrations Overall:(β highest = −1.91, 95% CI: −3.32, −0.50); for boys: (β highest = −1.89, 95%CI: −3.70, −0.50) and for girls: (β middle = −2.34, 95% CI: −4.61, −0.07)
Derakhshan et al. (2020) [60]	Positive association of BPA with TSH in newborns, especially in females [β [95% CI]: 0.04 (0.007)]
T4	
Sur et al. (2019) [62]	Negative correlation between BPA level and FT4 concentrations (r = −0.483, *p* < 0.02)
Wang et al. (2020) [57]	In mothers with higher BMI and higher BPA exposure, cord serum FT4 was 2.96 (95% CI 0.12–5.80) pmol/L higher in male newborns and 2.22 (95% CI 0.67–3.78) pmol/L higher in female newborns compared to those in the low tertile of BPA levels.
Guo et al. (2020) [55]	Maternal BPA concentrations were positively correlated with 1.00% (95%CI: 0.20%, 1.92%) increases in cord serum FT4 concentrations
Derakhshan et al. (2020) [60]	Lower FT4 concentration in childhood (β (95% CI): −0.11 (−0.21 to −0.01))
T3	
Fen Li et al. (2020) [54]	Children with the middle or highest tertile of BPA concentration also had lower TT3 (β highest = −0.05, 95%CI: −0.10,−0.01), FT3 (βhighest = −0.12, 95%CI: −0.22, −0.02; βmiddle = −0.11, 95% CI: −0.21, −0.01).
Jang et al., 2021 [59]	Gender specific effect between prenatal BPA and T3 concentrations (Boys *p* = 0.025; Girls *p* = 0.028)
Thyroid volume and multiple nodules risk	
Wang et al. (2015) [61]	Inverse association between urinary BPA concentration and thyroid volume (β = −0.033, 95% CI: −0.053, −0.013)
	Negative association with the risk of multiple nodules (OR = 0.78; 95% CI: 0.63, 0.97)

Abbreviations: TSH: thyroid stimulating hormone; BPA: bisphenol A; T4: thyroxine (T4); T3: Triiodothyronine; OR: Odds Ratio; CI: Confidence Interval.

## Data Availability

Not applicable.

## Supplementary Materials

The following are available online at https://www.mdpi.com/article/10.3390/nu14010168/s1, PRISMA-P (Preferred Reporting Items for Systematic review and Meta-Analysis Protocols) 2015 checklist.
